# Atypical placental site nodules: A retrospective case series

**DOI:** 10.1111/aogs.70160

**Published:** 2026-02-27

**Authors:** Victoria Louise Parker, Kamaljit Singh, Katie McDonald, Imran Jabbar, Matthew Christopher Winter, Julia Elizabeth Palmer

**Affiliations:** ^1^ Department of Gynaecological Oncology Sheffield Teaching Hospitals NHS Foundation Trust Sheffield UK; ^2^ School of Medicine and Population Health The University of Sheffield Sheffield UK; ^3^ Sheffield Trophoblastic Disease Centre, Sheffield Teaching Hospitals NHS Foundation Trust Sheffield UK; ^4^ South Yorkshire and Bassetlaw Pathology, Sheffield Teaching Hospitals NHS Foundation Trust Sheffield UK; ^5^ Sheffield Trophoblastic Disease Centre Sheffield UK

**Keywords:** atypical placental site nodule (APSN), epithelioid trophoblastic tumor (ETT), gestational trophoblastic disease, gestational trophoblastic neoplasia, placental site trophoblastic tumor (PSTT)

## Abstract

**Introduction:**

Atypical placental site nodule (APSN) is a rare diagnosis, representing remnants of a previous pregnancy and extravillous trophoblast tissue. These lesions are potential precursor lesions to rare forms of Gestational trophoblastic neoplasia (GTN). Recent data suggest up to a 15% risk of malignancy occurring either concurrently or manifesting within a few months of diagnosis. These patients are often young with future fertility considerations. Prognosis, treatment, and clinical follow‐up of APSN cases currently remain a matter of debate. This study aimed to address and explore some of these issues.

**Material and Methods:**

Retrospective case series analysis was performed between 1st January 2000 and 31st December 2023 at the Sheffield Trophoblastic Disease Centre, Sheffield, UK. Patients on conservative management were asked at routine follow‐up telephone consultations if they would consider a completion hysterectomy in light of the risk of progression to GTN.

**Results:**

Twenty‐two cases of APSN were registered, of which 10 (45%) received surgical management. Two (20%) cases were incidentally diagnosed following total abdominal hysterectomy (TAH) for other indications and eight (80%) had a TAH within twelve months of their initial diagnosis as part of primary management. None had histological evidence of GTN.

Of the twelve (55%) patients initially opting primarily for conservative management, three (25%) decided to have a TAH performed based on the current evidence for risk of malignant transformation, eight (67%) indicated they would have a TAH based on advice from the center, and one (8%) was uncertain. No patients were diagnosed with GTN.

**Conclusions:**

In this study, we found no evidence of malignant transformation in our patients, which conflicts with other published data conferring an 11%–14% risk of malignant transformation. An international consensus opinion needs to be reached within the Gestational trophoblastic community regarding the optimal advice, management, and follow‐up regimens for patients diagnosed with APSN.

## Graphical abstract

Retrospective series of 22 cases of atypical placental site nodule managed at a UK specialist trophoblastic disease center, revealing no cases of malignant conversion/Gestational trophoblastic neoplasia.

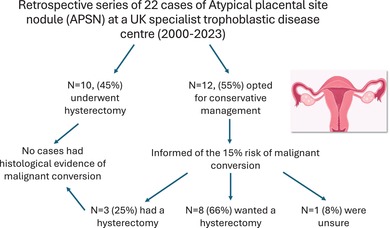

**Abbreviations**
APSN: atypical placental site nodule
CCTDC: Charing Cross Trophoblastic Disease Centre
CT: computerized tomography
EOTTD: European Organisation for the Treatment of Trophoblastic Diseases
ESGO: European Society of Gynaecological Oncology
ETT: epithelioid trophoblastic tumor
GCIG: gynecologic cancer InterGroup
GTN: gestational trophoblastic neoplasia
hCG: human chorionic gonadotrophin
ICE: integrated clinical environment
IMD: index of multiple deprivation
IQR: interquartile range
ISSTD: International Society for the Study of Trophoblastic Diseases
MRI: magnetic resonance imaging
PSN: placental site nodule
PSTT: placental site trophoblastic tumor
STDC: Sheffield Trophoblastic Disease Centre
TAH + BS: total abdominal hysterectomy + bilateral salpingectomy
WHO: World Health Organization

**Key message**

In a retrospective series of 22 cases of atypical placental site nodule at a UK specialist trophoblastic disease center, no cases of malignant transformation were detected at hysterectomy.

## 1 INTRODUCTION

Placental site nodules (PSN) occur in the reproductive age group, representing remnants of a previous pregnancy and its extravillous trophoblast. These benign lesions are usually incidentally detected during hysterectomy or in endometrial biopsies.^1^, ^2^, ^3^, ^4^, ^5^ Epithelioid trophoblastic tumors (ETT) and placental site trophoblastic tumors (PSTT) are rare forms of malignant Gestational trophoblastic neoplasia (GTN) diagnosed by histological examination of retained pregnancy tissue. They may occur many years after pregnancy, yet their presentation and behavior are not easy to predict.^6^ Hysterectomy ± chemotherapy is used to manage these tumors.^7^, ^8^, ^9^ In the United Kingdom, GTN is managed by two expert centers in Sheffield and London; Sheffield (STDC) and Charing Cross Trophoblastic Disease Centres (CCTDC).

In 1999, “Shih and Kurman” first described gestational atypical placental site nodules (APSN). These lesions have intermediate pathological features between PSN and ETT.^10^, ^11^ APSN was introduced into the World Health Organization (WHO) classification of female genital tract tumors in 2020.^12^ APSN may represent a concurrent or precursor lesion to ETT^13^ or PSTT.^14^, ^15^

The UK data from CCTDC has identified GTN in 14% of APSN cases and in 22% of those undergoing hysterectomy, with malignancy developing either concurrently or within 16 months of APSN diagnosis.^16^, ^17^ Conversely, preliminary data from Boston, USA, suggested a lower incidence (5%) of associated GTN at the time of hysterectomy.^18^ However, updated data are more in line with UK studies, revealing GTN in 11% of APSN cases and 15% of cases undergoing hysterectomy.^19^

**FIGURE 1:**
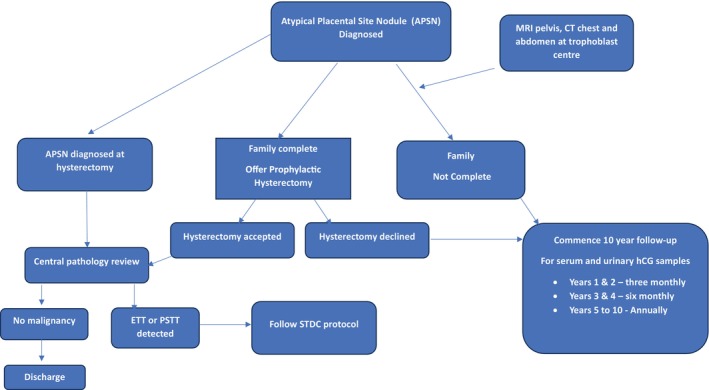
Protocol for atypical placental site nodule (APSN), Sheffield Trophoblastic Disease Centre, Sheffield, UK.

APSN cases are rare, hence their prognosis, treatment, and clinical follow‐up remain a matter of debate. At diagnosis, some expert centers advocate for patients to undergo radiological assessment with pelvic and head MRI (magnetic resonance imaging), chest and abdomen CT (computerized tomography) to assess for metastatic disease. Patients are followed up closely or offered prophylactic hysterectomy if their family is complete (Figure 1).^20^

Historically, STDC has not recommended hysterectomy following family completion. However, given the accumulating evidence conferring an 11%–14% risk of malignant transformation, this was introduced into standard practice in 2023. Following the change in practice, the aims of this study are to: (i) assess the attitude of patients to the offer of hysterectomy following completion of their families, and (ii) evaluate the outcomes of APSN cases managed by the STDC expert center.

## 2 MATERIAL AND METHODS

A retrospective case series was conducted at the STDC, Sheffield, UK. All patients registered in the clinical STDC database between 1st January 2000 and 31st December 2023 with a diagnosis of APSN were included. Patients with incomplete data or uncertainties regarding histological diagnosis were excluded.

The database was cross‐referenced, where appropriate, with patient notes and the Trust‐based integrated clinical environment (ICE) system (an established web‐based requesting and reporting service) to confirm patient demographic data, findings on imaging, and histological diagnoses.

Data were collated on age at registration, postcode, ethnicity, pre‐treatment human chorionic gonadotrophin (hCG) levels, histological diagnosis, and treatment modality (conservative management/surgery). Postcodes were utilized to calculate the index of multiple deprivation (IMD) quintile data using the English indices of deprivation 2019 postcode look‐up.^21^ Ethnicity was calculated using the Office for National Statistics 2021 census data, list of ethnic groups.^22^

As part of their routine follow‐up, patients on conservative management had telephone consultations with the nurse consultant at STDC and were informed of the reported data that APSN was potentially associated with a significant risk of malignant transformation to ETT or PSTT^17^ These patients were asked, in light of this new data, whether they would now prefer to proceed with a hysterectomy. The patient dataset was also updated with their pregnancy history following their APSN diagnosis.

Following internal validation and coding of data, the results were reported descriptively (n numbers and percentages). Since the study was performed in the context of a service review, ethical approval was not required.

## 3 RESULTS

During the study period, there were twenty‐two cases of APSN registered. The median age at diagnosis was 36 years with an interquartile range (IQR) of 8.3 years. The majority (86%) were of white ethnicity (eighteen White English, one White Other), and three (14%) were of black ethnicity (all Black African). The majority (64%) resided in areas of higher social deprivation (3rd to 5th IMD quintiles) (Table 1).

**TABLE 1:** Cases of atypical placental site nodule (APSN) at Sheffield trophoblastic disease center (STDC), Sheffield. UK.

Patient No	Year of diagnosis	Age at diagnosis	Ethnicity	hCG at diagnosis	Primary management	Histology at hysterectomy	Secondary management following telephone consult	Histology at hysterectomy
1	2010	25	White English	<1	Conservative	N/A	Conservative	N/A
2	2012	26	White English	<1	Conservative	N/A	Conservative	N/A
3	2014	26	White English	<1	Conservative	N/A	TAH ‐ 2024	No residual APSN
4	2016	38	White English	<1	TAH	No residual APSN	N/A	No residual APSN
5	2017	44	White English	<1	Diagnosed at TAH	APSN	N/A	APSN
6	2017	40	Black African	<1	Conservative	N/A	Conservative	N/A
7	2017	28	White English	<1	Conservative	N/A	Conservative	N/A
8	2017	36	White English	<1	Conservative	N/A	Conservative	N/A
9	2019	37	White English	<1	TAH	APSN	N/A	APSN
10	2019	36	White English	<1	TAH	No residual APSN	N/A	No residual APSN
11	2019	34	White Other	<1	Diagnosed at TAH	APSN	N/A	APSN
12	2020	41	Black African	<1	Conservative	N/A	Conservative	N/A
13	2020	35	White English	<1	TAH	APSN	N/A	APSN
14	2020	40	White English	<1	Conservative	N/A	Conservative	N/A
15	2021	37	White English	<1	TAH	No residual APSN	N/A	No residual APSN
16	2021	33	White English	<1	TAH	APSN	N/A	APSN
17	2022	37	White English	<1	Conservative	N/A	Conservative	Histology Awaited
18	2022	31	White English	<1	Conservative	N/A	TAH—2024	N/A
19	2023	43	Black African	<1	Conservative	N/A	Conservative	N/A
20	2023	36	White English	4	TAH	APSN	N/A	APSN
21	2024	33	White English	4	Conservative	N/A	TAH—2024	Histology Awaited
22	2024	28	White English	4	TAH	APSN	N/A	APSN

Abbreviation: TAH, total abdominal hysterectomy.

### 3.1 Primary surgical treatment

Ten patients (45%) underwent surgery in the form of total abdominal hysterectomy + bilateral salpingectomy (TAH + BS) with ovarian conservation as part of their primary treatment. Of the patients undergoing surgery, two (20%) had surgery for other indications and an incidental APSN diagnosis, and eight (80%) had surgery within twelve months of their initial APSN diagnosis, which was initially diagnosed following endometrial biopsy. Three (37%) patients had no residual APSN and five (63%) had residual APSN. No patients had histological evidence of either ETT or PSTT.

### 3.2 Primary conservative management

Of the twelve patients (55%) initially opting for conservative management, five (42%) had further pregnancies resulting in four term births, one miscarriage and one termination of pregnancy. Ten (83%) regarded their family as complete when questioned at routine follow‐up. Three patients (25%) decided to have a hysterectomy performed based on the risk of malignant transformation, at ten years, two years and one year post APSN diagnosis. Two patients (67%) had no residual APSN, while one had residual APSN (33%). Eight patients (67%) advised they would pursue a hysterectomy based on recommendations by the center, and one (8%) was uncertain.

Other than patient's fertility wishes, there were no differences between those choosing primary treatment in the form of hysterectomy and those opting for conservative management.

## 4 DISCUSSION

In this study, we found no evidence of malignant transformation in our patients, which conflicts with other published data conferring an 11%–14% risk of malignant transformation. The extrapolation of our study is limited by small patient numbers; the absence of retrospective pathological review to assess prior ETT and PSTT cases for concurrent APSN; and the absence of systematic data collection to explore reasons for conservative versus surgical management.

Assessing malignant transformation risk in APSN is difficult due to conflicting literature. However, the authors consider this study to be valid and generalizable to other care settings, given that STDC is one of only two UK expert GTN centers. UK patient information leaflets now state a 14%–15% risk. If we consider this in relation to the lifetime risk of other female cancers: breast cancer 1:7, endometrial cancer 1:39, ovarian cancer 1:56, and cervical cancer 1:130, a potential 15% risk (1:6.6) no longer seems that small.

APSN management is challenging, considering that malignant transformation may occur either concurrently or within 16 months of diagnosis.^16^ This raises questions regarding the extent of imaging at diagnosis and the optimal timing of surgery. Given the growing body of evidence regarding concurrent ETT or PSTT, baseline imaging at STDC now includes MRI of the pelvis and CT of the chest and abdomen to exclude the small possibility of already established metastatic disease. All patients who have completed childbearing are advised to undergo hysterectomy and are discharged if no ETT/PSTT is found.

The optimal timing of surgery remains unclear. PSTT and ETTs often grow slowly and may appear years after the index pregnancy,^7^, ^23^, ^24^, ^25^, ^26^ with poorer outcomes when the interval is ≥48 months.^23^ Therefore, should a hysterectomy be performed within 16 months of diagnosis, or <48 months from the index pregnancy? Genotyping APSN can help determine the interval from the causative pregnancy, which may not necessarily be the antecedent pregnancy.^27^

Further questions arise in those patients whose future fertility is important, including the long‐term and lifetime risk of malignant transformation, as case numbers are low and evidence is sparse. STDC traditionally performed a ten‐year follow‐up (see Figure 1) with serum and urinary hCG measurements and no higher imaging. However, as APSN, ETT, and PSTT may have normal hCG levels, it is unlikely that hCG screening is truly beneficial in this group of patients. Joint international EOTTD‐ESGO‐GCIG‐ISSTD guidelines for GTD^28^ recommend follow‐up with hCG measurements and imaging ± hysteroscopy, acknowledging that there is no evidence guiding the optimal length of follow‐up, type, or frequency of imaging. The benefits of surveillance must also be weighed against the risks of developing an additional cancer related to repeated CT imaging.

Given the rare nature of published APSN cases, with only 85 patients^16^, ^19^, ^29^, ^30^, ^31^, ^32^, ^33^, ^34^ (including our case series), an international pooled database, similar to that used for ETT, PSTT, and ultra‐high‐risk GTN^35^ would support guideline development. Further research could involve an international collaboration to perform expert retrospective pathology reviews of ETT and PSTT cases to identify coexistent APSN. This approach, however, would be subject to significant bias and unlikely to provide a true representation of risk, as many cases of APSN may have remained undiagnosed by nonspecialist pathologists in both endometrial biopsy and hysterectomy specimens, as the histological diagnosis of APSN remains challenging.^13^

## 5 CONCLUSION

In this study, we found no evidence of malignant transformation in our patients, which conflicts with other published data conferring an 11–14% risk of malignant transformation. An international expert consensus opinion needs to be reached regarding the optimal advice and management of APSN, in addition to follow‐up regimens when conservative management is chosen. International collaboration and pooling of data seem to be the obvious way forward and a subject for further discussion between both European and International trophoblastic societies.

## AUTHOR CONTRIBUTIONS

Victoria L. Parker and Julia E. Palmer were the principal authors and performed the analyses. Victoria L. Parker, Julia E. Palmer, and Kamaljit Singh collected data for the study. Victoria L. Parker, Julia E. Palmer, Imran Jabbar, Katie McDonald, and Matthew C. Winter performed the interpretation of data and revision of the manuscript.

## FUNDING INFORMATION

No funding was received for the conduct of this study.

## CONFLICT OF INTEREST STATEMENT

The authors declare there are no conflicts of interest.

## ETHICS STATEMENT

This project was conducted as a service review and did not constitute research requiring formal ethical approval from an institutional review board as per NHS Health Research Authority guidance. All data were handled in compliance with national and local Trust guidelines for clinical audit, ensuring the confidentiality of all individuals involved.

## Data Availability

The data that support the findings of this study are available from the corresponding author upon reasonable request.
